# Clinical course, treatment and management of generalised pustular psoriasis from a United Kingdom extension of a global Delphi panel

**DOI:** 10.1002/ski2.359

**Published:** 2024-03-16

**Authors:** Jonathan N. Barker, Gabrielle Becher, David A. Burden, Andrew E. Pink, Maria Zacharioudaki, Richard B. Warren

**Affiliations:** ^1^ St John's Institute of Dermatology Guy's Hospital Campus King's College London London UK; ^2^ Department of Dermatology West Ambulatory Care Hospital Glasgow UK; ^3^ Institute of Infection Immunity and Inflammation University of Glasgow Glasgow UK; ^4^ School of Infection and Immunity University of Glasgow Glasgow UK; ^5^ IQVIA Ltd Athens Greece; ^6^ Dermatology Centre Salford Royal NHS Foundation Trust Manchester UK; ^7^ NIHR Biomedical Research Centre The University of Manchester Manchester UK

## Abstract

This article presents the results of the UK extension of a previously conducted global Delphi panel on generalised pustular psoriasis (GPP). Five UK based dermatologists experienced in GPP management have expressed their level of agreement on 101 questionnaire statements addressing four aspects of GPP: clinical course and flare definition, diagnosis, treatment goals, and holistic management. Consensus was achieved for 89 of 101 statements (88%). Disagreement was detected on issues around the prognostic value of age, QoL assessment tools and the nature of comorbidities associated with GPP. Overall, the panelists corroborated the results of the global study and confirmed that the clinical algorithm derived from the global study is in accordance with the UK clinical practice.

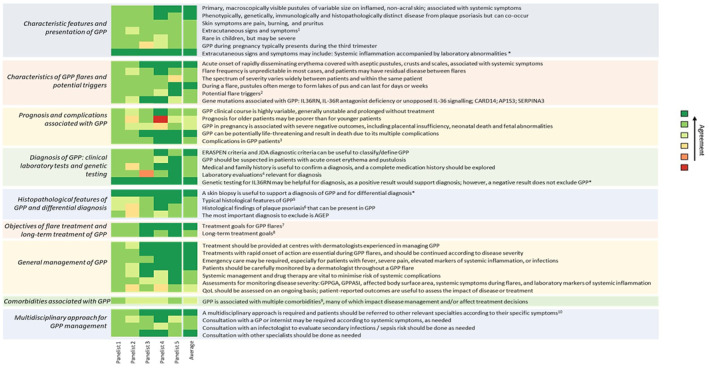

Dear Editor,

Generalised pustular psoriasis (GPP) is a rare, potentially life‐threatening, neutrophilic skin disease that is distinct from, Psoriasis vulgaris (Pv).^1^ Sparsity of clinical trials on GPP treatment options and lack of evidence‐based clinical guidelines pose important challenges on disease management. To address this issue, a global‐scale Delphi panel has recently been conducted among dermatologists experienced in GPP, focusing on four key domains of the disease: clinical course and flare definition, diagnosis, treatment goals, and holistic management.^2^ Based on statements where a clear consensus was achieved, a clinical management algorithm was developed.^2^ However, lack of United Kingdom (UK) representation in the global Delphi created the need for a UK‐focused extension to assess the generalisability of the findings in the UK healthcare system.

This extension involved a panel of five UK‐based dermatologists experienced in GPP management, who were asked to express their level of agreement on a Likert scale ranging from 1 (strong disagreement) to 7 (strong agreement), with the conclusions previously reached in the global study.

Consensus of agreement was defined as an attributed score of 5–7 from ≥80% of the panellists for a given statement and was achieved for 89 out of 101 statements (88%), confirming findings of the global Delphi. There was no statement with consensus of disagreement (score of 1–3 from ≥80% of the panellists). Statements with no consensus were discussed in a virtual workshop and were either rephrased in a way that more accurately reflects clinical reality (indicated with an asterisk in Figure 1), or remained unresolved, revealing gaps in the general understanding of GPP.

**FIGURE 1:**
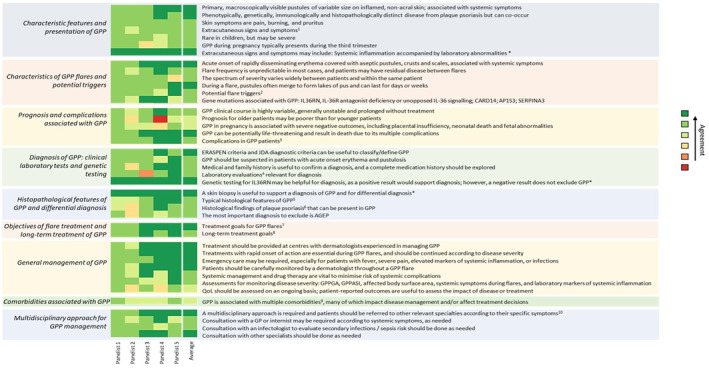
Statements on generalised pustular psoriasis with expert consensus. ^1^Fever, chills, malaise, arthritis, arthralgia, asthenia, fatigue, neutrophilic cholangitis, nail abnormalities, liver abnormalities, jaundice, oedema. ^2^Treatment, treatment discontinuation, bacterial or viral infections, pregnancy, stress, severe hypocalcemia secondary to hypoparathyroidism. ^3^Death (septic shock, cardiac failure), infections, superinfections, systemic/pulmonary capillary leak syndrome, septicaemia, sepsis, intestinal malabsorption, liver diseases, acute renal failure, heart failure, arthritis, aseptic purulent arthritis, myalgias, polymyalgia, polyarthralgia, bronchopneumonia, ARDS, iatrogenic complications. ^4^Complete blood cell count, elevated erythrocyte sedimentation rate, elevated C‐reactive protein, decreased albumin, decreased calcium and zinc, high blood urea nitrogen and creatinine if the patient is oligemic, elevated liver function enzymes in the case of liver damage, alkaline phosphatase, bilirubin. ^5^Neutrophilic subcorneal pustules histopathologically characterised by Kogoj's spongiform pustules, intense neutrophilic epidermal and dermal infiltration and forming pustules with subcorneal localisation as well as acanthosis, spongiosis and exocytosis in epidermis, superficial perivascular mononuclear cell infiltrations, intraepidermal pustules. ^6^Parakeratosis, acanthosis, hyperkeratosis, elongation of rete ridges, diminished stratum granulosum, capillary dilation of the papillary dermis. ^7^Rapid and sustained clearance of pustules, no fresh pustules, rapid and sustained clearance of inflammatory erythema, scaling, crust, complete and sustained clearance of skin lesions, improved control of consequences of systemic inflammation, rapid alleviation of systemic symptoms, reduction of pain, favourable safety profile. ^8^Prevention of new flares, sustained resolution of skin and systemic symptoms, no safety concerns with long‐term exposure, normalise health related quality of life. ^9^Obesity, diabetes mellitus, infections, psoriasis vulgaris (plaque psoriasis), psoriatic arthritis, polyarthritis, arthritis, hypertension, hormonal/metabolic conditions, auto‐immune conditions, liver disease, inflammatory skin conditions, subcutaneous tissue infections, septicaemia during hospitalisation, cardiovascular disease, ischaemic heart disease, chronic renal insufficiency, acute renal failure, depression, anxiety, fluid and electrolyte disorders. ^10^ARDS, capillary leak syndrome, cardiovascular impairment, arthritis. *These two statements reached consensus after being rephrased but were not assigned new scores. Their scores here are demonstrative and only represent the fact that they reached consensus. ERASPEN, European rare and severe psoriasis expert Network; GP, General Practitioner; GPPASI, generalised pustular psoriasis area and severity index; GPPGA, generalised pustular psoriasis physician global assessment; JDA, Japanese Dermatological Association.

Overall, UK experts corroborated the conclusions from the global Delphi panel, especially in the key areas, agreeing that, although they can co‐occur, GPP is phenotypically, genetically, and immunologically distinct from Pv, and can present with a wide range of cutaneous and extracutaneous manifestations. This complicates initial diagnosis, as it can often be hard to exclude other conditions such as acute generalised exanthematous pustulosis or infectious disease. However, laboratory evaluations and family history and histopathological considerations are helpful in confirming a GPP diagnosis. Unanimous consensus was achieved upon treatment goals; complete, rapid and sustained clearance of skin manifestations during flares, control and alleviation of systemic inflammation and related symptoms, prevention of new flares, long‐term safety and quality of life (QoL) improvement were deemed fundamental treatment requirements. Finally, panellists agreed on the key assessments for monitoring disease severity and acknowledged the importance of specialised care for GPP patients, as well as of a multidisciplinary approach for the optimal management of systemic complications and common comorbidities. There was uncertainty around the prognostic value of age, which can arguably be correlated to complication‐related morbidity but not necessarily to flare frequency. Furthermore, while panellists agreed on the value of QoL assessment, they argued that the existing standardised questionnaires do not account for the acute and paroxysmal nature of the condition, and thus may appear irrelevant and misleading. Finally, there was scepticism about the comorbidities possibly related to GPP, since there is yet limited high quality epidemiological evidence to support strong associations and eliminate confounding. A more detailed overview on the individual statements and their corresponding consensus levels is presented in Figure 1.

This study confirmed that the clinical algorithm derived by the global Delphi panel is generally in accordance with the UK clinical practice. Due to the rarity of the disease, the pool of GPP‐specialised physicians in the UK was limited, and thus a small sample size (*N* = 5) had been anticipated. However, Delphi panels constitute a suitable method for providing valuable insight on the most important issues regarding the core aspects of this rare and potentially fatal disease.^3^

This extension further draws the attention of the clinical community to the following objectives for further research: the need for a fit‐for‐purpose QoL tool, the role of age as a prognostic factor, and the nature of association of certain comorbidities with GPP. Overall, the view of the panellists on key characteristics of GPP flares, treatment goals, diagnosis, complications, multidisciplinary management of the disease and GPP being distinct from Pv was consistent with the findings of the global Delphi panel.

## CONFLICT OF INTEREST STATEMENT

Burden declares paid consulting activities for AbbVie, Almirall, Boehringer Ingelheim, Celgene, Eli Lilly, Janssen, LEO Pharma, Novartis and UCB. Pink reports personal fees and nonfinancial support from LEO Pharma, Novartis, and UCB and personal fees from AbbVie, Almirall, Amgen, BMS, Boehringer, Janssen, La Roche‐Posay, Lilly, Pfizer, and Sanofi. Warren has received research grants or consulting fees from AbbVie, Almirall, Amgen, Arena, Astellas, Avillion, Boehringer Ingelheim, Bristol Myers Squibb, Celgene, DiCE, Eli Lilly, GSK, Janssen, LEO Pharma, Medac, Novartis, Pfizer, Sanofi, Sun Pharma, UCB, and UNION. He is supported by the Manchester NIHR Biomedical Research Centre. Becher reported personal fees from UCB, AbbVie, Almirall, Boehringer Ingelheim, UCB, Novartis, Janssen, and Eli Lilly outside the submitted work. Barker has received honoraria and/or research grants from AbbVie, Almirall, Amgen, Boehringer Ingelheim, Bristol Myers Squibb, Celgene, Janssen, LEO Pharma, Lilly, Novartis, Samsung and Sun Pharma. Li is an employee of Boehringer Ingelheim Ltd. Zacharioudaki is an employee of IQVIA.

## AUTHOR CONTRIBUTIONS

**Jonathan N. Barker**: Investigation (equal); supervision (equal); writing – review & editing (equal). **Gabrielle Becher**: Investigation (equal); supervision (equal); writing – review & editing (equal). **David A. Burden**: Investigation (equal); supervision (equal); writing – review & editing (equal). **Andrew E. Pink**: Investigation (equal); supervision (equal); writing – review & editing (equal). **Maria Zacharioudaki**: Data curation (lead); formal analysis (lead); writing – original draft (equal); writing – review & editing (equal). **Richard B. Warren**: Investigation (equal); supervision (equal); writing – review & editing (equal).

## ETHICS STATEMENT

Not applicable.

## Data Availability

The data underlying this article will be shared on reasonable request to the corresponding author.
